# Differences in Clinical Outcomes According to Weaning Classifications in Medical Intensive Care Units

**DOI:** 10.1371/journal.pone.0122810

**Published:** 2015-04-15

**Authors:** Byeong Ho Jeong, Myeong Gyun Ko, Jimyoung Nam, Hongseok Yoo, Chi Ryang Chung, Gee Young Suh, Kyeongman Jeon

**Affiliations:** 1 Division of Pulmonary and Critical Care Medicine, Department of Medicine, Samsung Medical Center, Sungkyunkwan University School of Medicine, Seoul, Korea; 2 Intensive Care Unit Nursing Department, Samsung Medical Center, Sungkyunkwan University School of Medicine, Seoul, Korea; 3 Department of Critical Care Medicine, Samsung Medical Center, Sungkyunkwan University School of Medicine, Seoul, Korea; Azienda Ospedaliero-Universitaria Careggi, ITALY

## Abstract

**Background:**

Although the weaning classification based on the difficulty and duration of the weaning process has been evaluated in the different type of intensive care units (ICUs), little is known about clinical outcomes and validity among the three groups in medical ICU. The objectives of this study were to evaluate the clinical relevance of weaning classification and its association with hospital mortality in a medical ICU with a protocol-based weaning program.

**Methods:**

All consecutive patients admitted to the medical ICU and requiring mechanical ventilation (MV) for more than 24 hours were prospectively registered and screened for weaning readiness by a standardized weaning program between July 2010 and June 2013. Baseline characteristics and outcomes were compared across weaning classifications.

**Results:**

During the study period, a total of 680 patients were weaned according to the standardized weaning protocol. Of these, 457 (67%) were classified as simple weaning, 136 (20%) as difficult weaning, and 87 (13%) as prolonged weaning. Ventilator-free days within 28 days decreased significantly from simple to difficult to prolonged weaning groups (*P* < 0.001, test for trends). In addition, reintubation within 48 hours after extubation (*P* < 0.001) and need for tracheostomy during the weaning process (*P* < 0.001) increased significantly across weaning groups. Finally, ICU (*P* < 0.001), post-ICU (*P* = 0.001), and hospital (*P* < 0.001) mortalities significantly increased across weaning groups. In a multiple logistic regression model, prolonged weaning but not difficult weaning was still independently associated with ICU (adjusted OR 8.265, 95% CI 3.484–19.605, *P* < 0.001), and post-ICU (adjusted OR 3.180, 95% CI 1.349–7.497, *P* = 0.005), and hospital (adjusted OR 5.528, 95% CI 2.801–10.910, *P* < 0.001) mortalities.

**Conclusions:**

Weaning classification based on the difficulty and duration of the weaning process may provide prognostic information for mechanically ventilated patients who undergo the weaning process.

## Introduction

Weaning from mechanical ventilation (MV) is a major issue in intensive care units (ICUs) [1]. The weaning process comprises at least 40% of the total duration of MV [2], and prolonged MV is associated with significant morbidity and mortality [3]. Therefore, minimizing the duration of MV is an important consideration for clinicians who care for critically ill patients, and weaning from MV should be considered as soon as possible. A recent meta-analysis revealed that in most trials, protocol-based weaning has been shown to reduce duration of MV, weaning, and ICU length of stay [4]. However, approximately 15% of patients receiving MV require a prolonged process of weaning and experience higher mortality [5].

An international consensus conference on weaning from MV in 2005 proposed that weaning be categorized into three groups (simple, difficult, and prolonged) based on the difficulty and duration of the weaning process [6]. However, this classification was based on expert opinion rather than rigorous analysis of a cohort of ventilated patients. Therefore, it was noted that further research with careful testing and analysis of groups of patients undergoing weaning are needed [6]. Although this classification has been evaluated in different types of ICUs [7–10], little is known about clinical outcomes and validity among the three groups in medical ICUs with a relatively high proportion of weaning difficulty.

The objectives of this study were to evaluate the clinical relevance of the weaning classification and its association with clinical outcomes in a medical ICU with a protocol-based weaning program.

## Materials and Methods

This retrospective analysis of prospectively collected data was conducted in the medical ICU at Samsung Medical Center (a 1,960-bed, university-affiliated, tertiary referral hospital in Seoul, South Korea), which included 28 beds between July 2010 and June 2013. The study was approved by the Institutional Review Board of Samsung Medical Center to review and publish information obtained from patient records (approval number 2013-05-043). The institutional review board waived the need for written informed consent from the patient because of the observational nature of the study. In addition, patients’ information was anonymized and de-identified prior to analysis.

### Study Population

**Fig 1:**
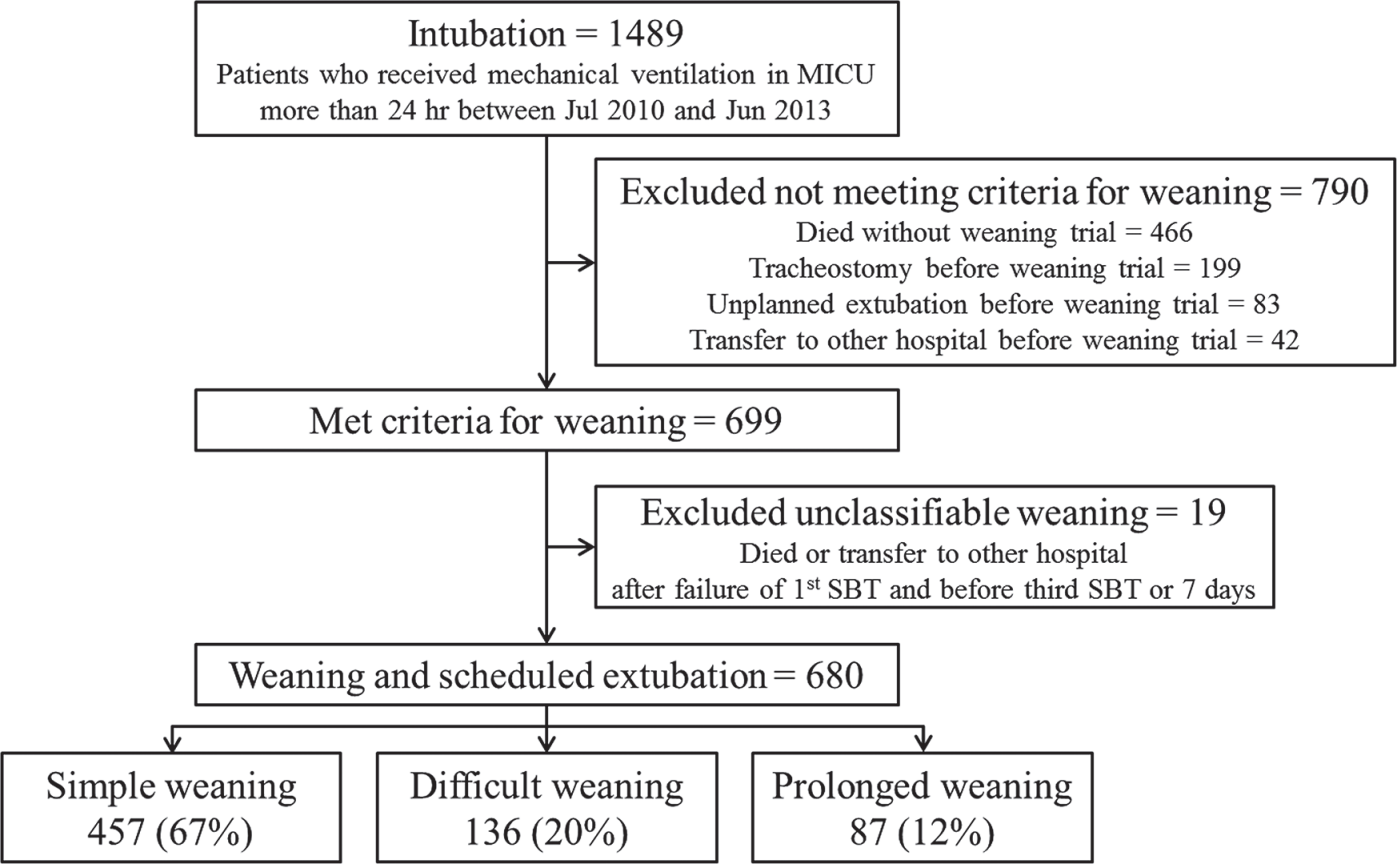
Flow chart of inclusion and exclusion criteria. MICU, medical intensive care unit; SBT, spontaneous breathing trial.

Over the study period, all consecutive patients admitted to the medical ICU and requiring MV for more than 24 hours were prospectively registered and screened for weaning readiness by a standardized weaning program adapted from recent recommendations [6]. For the purpose of this study, we selected only patients who underwent a spontaneous breathing trial (SBT) for weaning from MV at least once. Therefore, we excluded patients who died, who were transferred to other hospitals before they were ready to wean, or who experienced unplanned extubation or tracheostomy before the weaning process. Finally, we included all consecutive medical ICU patients who were mechanically ventilated for more than 24 hours and weaned according to the protocol without unplanned extubation or tracheostomy before the weaning process (Fig 1). If a patient was re-admitted to the ICU for MV support during the same episode of hospital admission, only the first weaning episode was included in analysis. Multiple ICU visits during different episodes of hospital admission, however, were enrolled separately.

### Standardized Weaning Process

**Fig 2:**
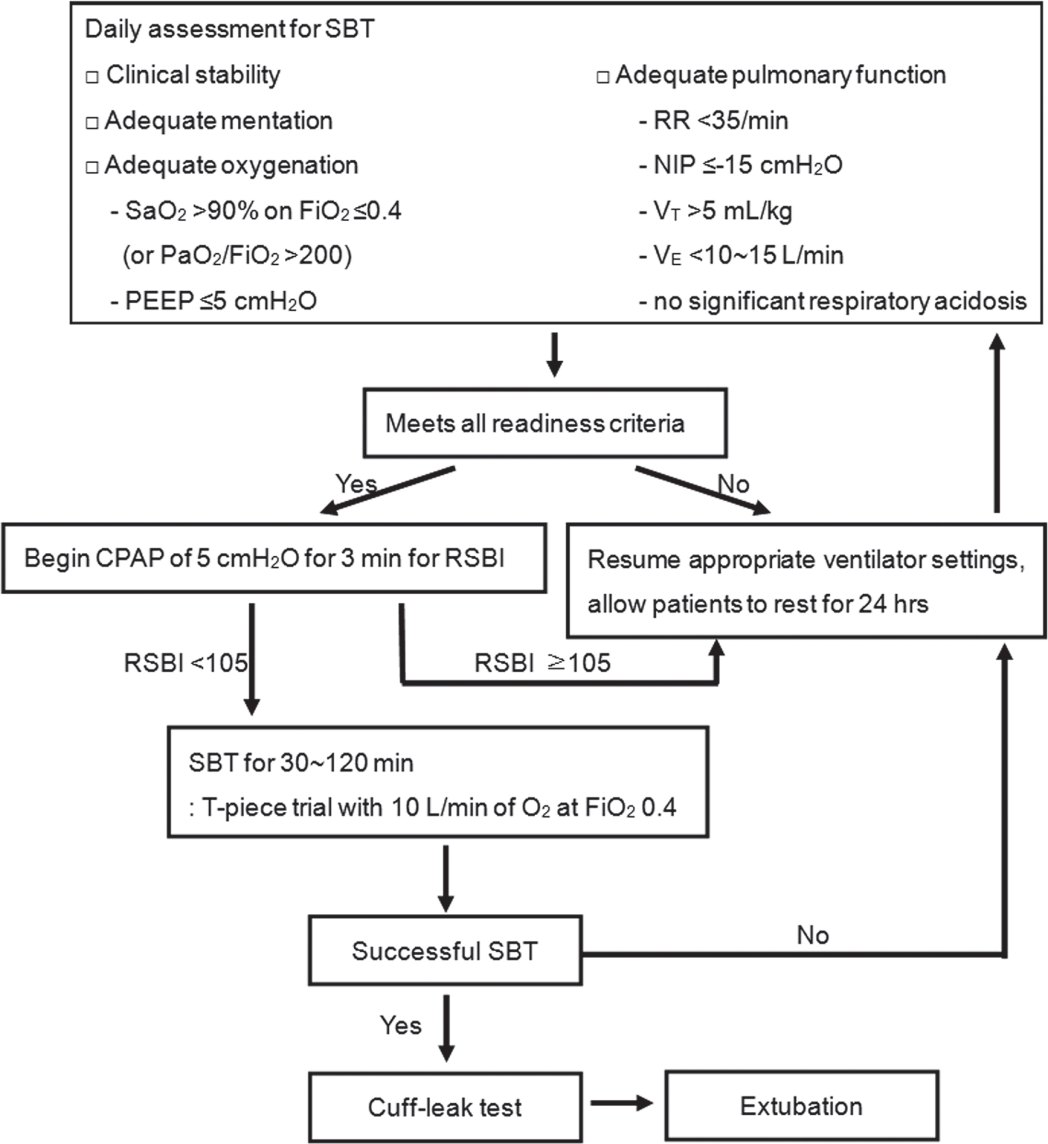
Weaning protocol applied to this study. SBT, spontaneous breath trial; PEEP, positive end expiratory pressure; RR, respiratory rate; NIP, negative inspiratory pressure; V_T_, tidal volume; V_E_, minute ventilation; CPAP, continuous positive airway pressure; RSBI, rapid shallow breathing index. * Negative inspiratory pressure (NIP) is the lowest pressure generated during a forceful inspiratory effort against an occluded airway, which is determined by occluding the ventilator’s inspiratory port at the end of expiration for 20 seconds and reading the maximum negative pressure registered on the ventilator’s pressure manometer.

A specific protocol-based weaning program based on recent recommendations [6] has been implemented in our center since 2010 (Fig 2). Respiratory care practitioners (RCPs), registered nurses specializing in respiratory care, screened patients daily for weaning readiness according to the criteria adapted from recent recommendations [6] (S1 Table). The weaning protocol was initiated when the Richimond Agitation-Sedation Scale score was 0 to -1 after sedation vacation in the morning. The patients who fulfilled these criteria were assessed for the likelihood of a successful SBT with a calculation of the rapid shallow breathing index (RSBI, the ratio of respiratory frequency to tidal volume) on continuous positive airway pressure (CPAP) of 5 cmH_2_O for 3 minutes [11]. If the RSBI was less than 105, the patients underwent a SBT as a diagnostic test to assess the likelihood of successful weaning. The initial SBT consisted of breathing with a T-piece at 9–10 L/min with 40% inspiratory oxygen fraction (FiO_2_) and lasted 30 min. When a patient successfully passed the SBT according to the criteria (S2 Table), the patient underwent the cuff-leak test for extubation readiness as described by Krinner et al. [12]. When a patient failed the cuff-leak test (absolute volume < 110 mL or 15% of exhaled volume), the patient received intravenous infusion of methylprednisolone every 6 hours over 24 hours before extubation [13] and then was extubated without repeated cuff-leak test. When a patient successfully passed the cuff-leak test, extubation proceeded immediately. If a patient failed the SBT, MV was restarted, and the team reviewed the possible reversible etiologies for the failure. When the patient was again ready for weaning, the SBT was repeated for 120 min on the next day. Extubation failure was defined as the need for reinstitution of ventilatory support within 48 hours of planned extubation based on criteria adapted from recent recommendations (S3 Table).

### Classification of Patients According to the Weaning Process

Patients were classified into one of three groups based on the difficulty and duration of the weaning process as follows: simple weaning, patients who proceed from initiation of weaning to successful extubation on the first attempt without difficulty; difficult weaning, patients who fail initial weaning and require up to three SBTs or as long as 7 days from the first SBT to achieve successful weaning; or prolonged weaning, patients who require more than three SBTs or >7 days of weaning process after the first SBT [6].

### Data Collection

The following information was collected on each registered patient: demographic data, the primary reasons for ICU admission, Simplified Acute Physiology Score 3 (SAPS 3) [14] and Sequential Organ Failure Assessment (SOFA) [15] at the time of ICU admission, day of initiating MV, and indications for MV. The time from initiating MV to first SBT, SOFA score on the day of first SBT, weaning indices (respiratory rate, maximum negative inspiratory pressure, PaO_2_/FiO_2_ ratios, and RSBI), results of arterial blood gas analysis (ABGA) at the beginning and end of first SBT, and weaning results were evaluated prospectively. The following data and events were assessed as clinical outcomes: ventilator-free days within 28 days, reintubation within 48 hours after extubation, need for tracheostomy during weaning process after first SBT, length of stay in ICU and hospital, mortality in ICU and hospital, mortalities in ICU, post-ICU, and hospital, and type of hospital discharge.

### Statistical Analyses

The data are presented as medians and interquartile ranges (IQR) for continuous variables and as numbers and percentages for categorical variables. The Jonckheere-Terpstra test for continuous variables [16] and the Mantel–Haenszel test for categorical variables [17] were used to compare baseline characteristics and outcomes across weaning classifications.

Additionally, the data were then compared between survivors and non-survivors using the Mann-Whitney U test for continuous variables and the Chi-square tests or Fisher’s exact test for categorical variables. To adjust for potential confounding factors in the association of weaning classification with ICU, post-ICU, and hospital mortalities, multiple logistic regression analysis was used. Simple weaning was defined as the reference category. Three models were constructed: the first model was adjusted for demographic data, such as age, sex, and underlying disease; the second model was additionally adjusted for the severity of illness on ICU admission (SAPS 3 and SOFA) and causes of respiratory failure; and the third model additionally included the data on the day of first SBT, such as SOFA score, duration of MV before first SBT attempt, weaning indices, and results of ABGA at the beginning and end of first SBT. Data are presented as adjusted odds ratios (ORs) with 95% confidence intervals (CI). If there were multiple comparisons, we corrected the *P* value and CI using the Bonferroni method. All tests were two-sided, and a *P* value of < 0.05 was considered significant. Data were analyzed using PASW Statistics 18 (SPSS Inc, Chicago, Illinois).

## Results

During the study period, 1,489 consecutive patients requiring MV support were admitted to the medical ICU. Of these patients, 790 were excluded according to the exclusion criteria: 466 died, 42 were transferred to another hospital before they were ready to wean, 83 underwent unplanned extubation, and 199 had a tracheostomy before the weaning process. In addition, patients who failed an initial weaning trial and were transferred to another hospital during the weaning process were excluded because they could not be classified into difficult or prolonged weaning (n = 19). Finally, 680 patients who were weaned according to the standardized weaning protocol were included in this study (Fig 1); of these, 457 (67%) were classified in the simple weaning group, 136 (20%) in the difficult weaning group, and 87 (13%) in the prolonged weaning group.

Comparisons of baseline characteristics based on weaning classification are presented in Table 1. The proportion of patients with malignancy or neurologic disease increased with difficulty of weaning. Across weaning classification from simple to prolonged weaning groups, pneumonia was increased (*P* = 0.029) and pulmonary edema was decreased (*P* = 0.021) as a cause of respiratory failure.

**Table 1:** Baseline characteristics of patients according to weaning classification.

	Total (N = 680)	Simple weaning (n = 457, 67%)	Difficult weaning (n = 136, 20%)	Prolonged weaning (n = 87, 13%)	*P* value for trend test
Age, years	67 (53–74)	65 (53–73)	68 (55–75)	68 (53–75)	0.060
Sex, male	474 (69.7)	320 (70.0)	90 (66.2)	64 (73.6)	0.822
Underlying disease					
Malignancy	354 (52.1)	221 (48.4)	74 (54.4)	59 (67.8)	0.001
Respiratory	191 (28.1)	122 (26.7)	40 (29.4)	29 (33.3)	0.189
Cardiovascular	94 (13.8)	70 (15.3)	15 (11.0)	9 (10.5)	0.123
Neurologic	92 (13.5)	54 (11.8)	20 (14.7)	18 (20.7)	0.027
Genitourinary	86 (12.6)	63 (13.8)	15 (11.0)	8 (9.2)	0.183
Gastrointestinal	67 (9.9)	47 (10.3)	17 (12.5)	3 (3.4)	0.172
Cause of respiratory failure					
Pneumonia	251 (36.9)	160 (35.0)	48 (35.3)	43 (49.4)	0.029
Extrapulmonary sepsis	125 (18.4)	90 (19.7)	26 (19.1)	9 (10.3)	0.070
ARDS	61 (9.0)	35 (7.7)	16 (11.8)	10 (11.5)	0.122
Pulmonary edema	51 (7.5)	41 (9.0)	8 (5.9)	2 (2.3)	0.021
CPR	41 (6.0)	31 (6.8)	8 (5.9)	2 (2.3)	0.129
Ventilation failure	38 (5.6)	24 (5.3)	10 (7.4)	4 (4.6)	0.907
Coma	33 (4.9)	20 (4.4)	6 (4.4)	7 (8.0)	0.258
Exacerbation of ILD	17 (2.5)	13 (2.8)	2 (1.5)	2 (2.3)	0.613
Central airway obstruction	15 (2.2)	11 (2.4)	2 (1.5)	2 (2.3)	0.858
Miscellaneous	48 (7.1)	32 (7.0)	10 (7.4)	6 (6.9)	0.980
SAPS 3 on ICU admission	61 (51–74)	61 (50–73)	64 (54–76)	62 (51–76)	0.042
SOFA score on ICU admission	7 (5–11)	7 (5–11)	7 (5–11)	8 (4–11)	0.983

ARDS, acute respiratory distress syndrome; CPR, cardiopulmonary resuscitation; ILD, interstitial lung disease; SAPS, simplified acute physiology score; ICU, intensive care unit; SOFA, sequential organ failure assessment.

Data presented as medians and interquartile ranges (IQR), numbers (percentage).

Data on the day of first SBT are presented in Table 2. Duration of MV before first SBT increased significantly across weaning groups (*P* < 0.001). Severity of organ failure on the day of first SBT also increased significantly across weaning groups (*P* < 0.001). Weaning indices, such as respiration rate (*P* < 0.001) and PaO_2_/FiO_2_ ratios (*P* < 0.001), were worst in the prolonged weaning group; however, RSBI did not vary between weaning groups (*P* = 0.101). Although ABGA results at the beginning of first SBT were not different between weaning groups, ABGA results at the end of first SBT were different: lower pH (*P* = 0.004), higher PaCO_2_ (*P* = 0.016), and lower PaO_2_/FiO_2_ ratio (*P* < 0.001) across weaning classification from simple to prolonged weaning groups.

**Table 2:** Data on the day of first spontaneous breathing trial (SBT) for weaning from mechanical ventilation (MV).

	Total (N = 680)	Simple weaning (n = 457, 67%)	Difficult weaning (n = 136, 20%)	Prolonged weaning (n = 87, 13%)	*P* value for trend test
Duration of MV before first SBT, days	3 (2–6)	3 (2–5)	3 (2–6)	4 (2–8)	<0.001
SOFA score on the day of first SBT	5 (3–7)	4 (3–7)	6 (3–8)	6 (4–8)	<0.001
ABGA results at the beginning of first SBT					
pH	7.46 (7.42–7.50)	7.46 (7.43–7.50)	7.46 (7.42–7.50)	7.46 (7.41–7.50)	0.485
PaCO_2_, mmHg	36.6 (31.5–41.8)	36.3 (31.7–41.3)	36.6 (31.3–42.3)	38.7 (31.2–44.1)	0.255
Weaning indices on the day of first SBT					
RR, /min	18 (15–22)	18 (14–22)	18 (15–23)	20 (16–24)	<0.001
NIP, cmH_2_O	-21 (-17–-27)	-21 (-17–-27)	-21 (-17–-26)	-22 (-20–-29)	0.108
PaO_2_/FiO_2_ ratio	264 (213–340)	288 (228–357)	234 (192–318)	233 (190–270)	<0.001
RSBI	49 (34–68)	47 (32–67)	51 (39–70)	50 (40–71)	0.101
ABGA results at the end of first SBT					
pH	7.46 (7.42–7.50)	7.46 (7.43–7.50)	7.46 (7.41–7.49)	7.45 (7.39–7.49)	0.004
PaCO_2_, mmHg	36.1 (30.9–41.4)	35.7 (30.8–40.8)	36.7 (31.6–43.0)	38.8 (30.5–43.8)	0.016
PaO_2_/FiO_2_ ratio	217 (173–296)	236 (190–324)	183 (147–251)	172 (144–222)	<0.001

SOFA, sequential organ failure assessment; ABGA, arterial blood gas analysis; PaCO_2_, arterial carbon dioxide tension; RR, respiratory rate; NIP, negative inspiratory pressure; PaO_2_, arterial oxygen tension; FiO_2_, inspiratory oxygen fraction; RSBI, rapid shallow breathing index.

Data presented as medians and interquartile ranges (IQR), numbers (percentage).

Table 3 shows the comparison of clinical outcomes across weaning groups. Ventilator-free days within 28 days decreased significantly across weaning groups (*P* < 0.001). The proportion of reintubation within 48 hours after extubation (*P* < 0.001) and the need for tracheostomy during the weaning process (*P* < 0.001) increased significantly across weaning groups. Length of stay in ICU and hospital were also significantly longer across weaning groups (*P* < 0.001). In addition to increased mortality in the ICU (*P* < 0.001) and hospital (*P* < 0.001), post-ICU mortality during hospitalization was also significantly higher across weaning groups (*P* = 0.001). In survivors, discharge to home was greater in the simple weaning group, while hopeless discharge increased across weaning groups (*P* < 0.001, *P* = 0.008, respectively).

**Table 3:** Clinical outcomes according to weaning classification.

	Total (N = 680)	Simple weaning (n = 457, 67%)	Difficult weaning (n = 136, 20%)	Prolonged weaning (n = 87, 13%)	*P* value for trend test
Ventilator-free days within 28 days	24 (20–26)	25 (23–26)	22 (19–24)	9 (0–16)	<0.001
Reintubation within 48hrs after extubation	56 (8.2)	0	28 (20.6)	28 (32.2)	<0.001
Tracheostomy	121 (17.8)	32 (7.0)	33 (24.3)	56 (64.4)	<0.001
ICU stay, days	7 (4–13)	5 (3–9)	10 (6–14)	21 (15–32)	<0.001
ICU mortality	62 (9.1)	21 (4.6)	9 (6.6)	32 (36.8)	<0.001
Post-ICU mortality in ICU survivors	124/618 (20.1)	75/436 (17.2)	30/127 (23.6)	19/55 (34.5)	0.001
Hospital stay, days	26 (16–50)	23 (14–43)	30 (20–51)	42 (30–73)	<0.001
Hospital mortality	186 (27.4)	96 (21.1)	39 (28.7)	51 (58.6)	<0.001
Type of discharge					
Home	361 (53.1)	282 (61.8)	68 (50.0)	11 (12.6)	<0.001
Other hospital	118 (17.4)	73 (16.0)	25 (18.4)	20 (23.0)	0.110
Hopeless	15 (2.2)	6 (1.3)	4 (2.9)	5 (5.7)	0.008
Death	186 (27.4)	96 (21.1)	39 (28.7)	51 (58.6)	<0.001

ICU, intensive care unit.

Data presented as medians and interquartile ranges (IQR), numbers (percentage).

Comparisons of baseline characteristics and weaning classification between survivors and non-survivors during hospitalization are presented in Table 4. Underlying malignancy, severity of illness on ICU admission, duration of MV before first SBT, and SOFA score on the day of first SBT were significantly associated with ICU, post-ICU, and hospital mortalities. Simple weaning was more common while prolonged weaning was less common in survivors compared to non-survivors.

**Table 4:** Comparison of baseline characteristics and weaning classification between survivors and non-survivors during hospitalization.

	ICU mortality (n = 680)			Post-ICU mortality in ICU survivors (n = 618)			Hospital mortality (n = 680)		
	Survivors (n = 618)	Non-survivors (n = 62)	*P*	Survivors (n = 494)	Non-survivors (n = 124)	*P*	Survivors (n = 494)	Non-survivors (n = 186)	*P*
Age, years	66 (54–74)	67 (53–72)	0.861	65 (53–73)	69 (56–75)	0.062	65 (53–73)	68 (56–74)	0.129
Sex, male	427 (69.1)	47 (75.8)	0.273	336 (68.0)	91 (73.4)	0.247	336 (68.0)	138 (74.2)	0.118
Underlying disease									
Malignancy	310 (50.2)	44 (71.0)	0.002	223 (45.1)	87 (70.2)	<0.001	223 (45.1)	131 (70.4)	<0.001
Respiratory	172 (27.8)	19 (30.6)	0.638	144 (29.1)	28 (22.6)	0.144	144 (29.1)	47 (25.3)	0.315
Cardiovascular	87 (14.1)	7 (11.3)	0.544	73 (14.8)	14 (11.3)	0.318	73 (14.8)	21 (11.3)	0.240
Neurologic	82 (13.3)	10 (16.1)	0.530	71 (14.4)	11 (8.9)	0.106	71 (14.4)	21 (11.3)	0.295
Genitourinary	83 (13.4)	3 (4.8)	0.052	69 (14.0)	14 (11.3)	0.434	69 (14.0)	17 (9.1)	0.091
Gastrointestinal	59 (9.5)	8 (12.9)	0.398	42 (8.5)	17 (13.7)	0.078	42 (8.5)	25 (13.4)	0.054
Cause of respiratory failure									
Pneumonia	224 (36.2)	27 (43.5)	0.256	180 (36.4)	44 (35.5)	0.843	180 (36.4)	71 (38.2)	0.676
Extrapulmonary sepsis	112 (18.1)	13 (21.0)	0.581	84 (17.0)	28 (22.6)	0.150	84 (17.0)	41 (22.0)	0.130
ARDS	56 (9.1)	5 (8.1)	0.793	42 (8.5)	14 (11.3)	0.334	42 (8.5)	19 (10.2)	0.486
Pulmonary edema	48 (7.8)	3 (4.8)	0.611	43 (8.7)	5 (4.0)	0.082	43 (8.7)	8 (4.3)	0.052
CPR	40 (6.5)	1 (1.6)	0.164	32 (6.5)	8 (6.5)	0.992	32 (6.5)	9 (4.8)	0.423
Ventilation failure	36 (5.8)	2 (3.2)	0.566	28 (5.7)	8 (6.5)	0.739	28 (5.7)	10 (5.4)	0.883
Coma	29 (4.7)	4 (6.5)	0.531	26 (5.3)	3 (2.4)	0.181	26 (5.3)	7 (3.8)	0.417
Exacerbation of ILD	15 (2.4)	2 (3.2)	0.662	13 (2.6)	2 (1.6)	0.747	13 (2.6)	4 (2.2)	1.000
Central airway obstruction	13 (2.1)	2 (3.2)	0.639	11 (2.2)	2 (1.6)	1.000	11 (2.2)	4 (2.2)	1.000
Miscellaneous	45 (7.3)	3 (4.8)	0.609	35 (7.1)	10 (8.1)	0.707	35 (7.1)	13 (7.0)	0.965
SAPS 3 on ICU admission	61 (50–73)	66 (55–76)	0.046	60 (49–71)	67 (57–81)	<0.001	60 (49–71)	67 (56–79)	<0.001
SOFA score on ICU admission	7 (5–11)	9 (5–11)	0.062	7 (4–10)	9 (6–12)	0.001	7 (4–10)	9 (6–11)	<0.001
Duration of MV before first SBT, days	3 (2–5)	4 (2–7)	0.002	3 (2–5)	3 (2–6)	0.035	3 (2–5)	4 (2–7)	0.001
SOFA score on the day of first SBT	5 (3–7)	6 (4–9)	<0.001	4 (3–7)	5 (3–8)	<0.001	4 (3–7)	6 (4–8)	<0.001
ABGA at the beginning of first SBT									
pH	7.46 (7.42–7.50)	7.46 (7.42–7.50)	0.750	7.46 (7.42–7.50)	7.46 (7.43–7.50)	0.090	7.46 (7.42–7.50)	7.46 (7.43–7.50)	0.107
PaCO_2_, mmHg	36.6 (31.9–41.9)	36.8 (30.3–40.2)	0.247	36.8 (31.9–42.1)	35.9 (30.8–41.1)	0.126	36.8 (31.9–42.1)	36.0 (30.6–41.0)	0.058
Weaning index									
RR, /min	18 (15–22)	20 (16–25)	0.005	18 (15–22)	18 (15–22)	0.645	18 (15–22)	19 (15–23)	0.066
NIP, cmH_2_O	-21 (-27–-17)	-25 (-31–-19)	0.005	-21 (-27–-17)	-20 (-24–-17)	0.049	-21 (-17–-27)	-21 (-18–-27)	0.868
PaO_2_/FiO_2_ ratio	270 (216–343)	228 (191–308)	0.001	264 (215–338)	294 (230–376)	0.093	264 (215–338)	262 (209–349)	0.807
RSBI	49 (33–68)	50 (37–68)	0.517	48 (33–68)	50 (35–66)	0.840	48 (33–68)	50 (35–67)	0.594
ABGA at the end of first SBT									
pH	7.46 (7.42–7.49)	7.45 (7.41–7.50)	0.527	7.46 (7.42–7.49)	7.46 (7.43–7.51)	0.267	7.46 (7.42–7.49)	7.46 (7.42–7.50)	0.529
PaCO_2_, mmHg	36.1 (31.1–41.3)	37.7 (29.5–43.1)	0.881	36.4 (31.4–41.7)	34.4 (30.0–40.3)	0.025	36.4 (31.4–41.7)	34.9 (29.7–40.7)	0.048
PaO_2_/FiO_2_ ratio	222 (177–302)	178 (148–224)	<0.001	222 (176–305)	220 (179–293)	0.955	222 (176–305)	204 (165–274)	0.037
Weaning classification			<0.001			0.001			<0.001
Simple	436 (70.6)	21 (33.9)		361 (73.1)	75 (60.5)		361 (73.1)	96 (51.6)	
Difficult	127 (20.6)	9 (14.5)		97 (19.6)	30 (24.2)		97 (19.6)	39 (21.0)	
Prolonged	55 (8.9)	32 (51.6)		36 (7.3)	19 (15.3)		36 (7.3)	51 (27.4)	

ARDS, acute respiratory distress syndrome; CPR, cardiopulmonary resuscitation; ILD, interstitial lung disease; SAPS, simplified acute physiology score; ICU, intensive care unit; SOFA, sequential organ failure assessment; MV, mechanical ventilation; SBT, spontaneous breath trial; ABGA, arterial blood gas analysis; RR, respiratory rate; NIP, negative inspiratory pressure; PaO_2_, arterial oxygen tension; FiO_2_, inspiratory oxygen fraction; RSBI, rapid shallow breathing index, PaCO_2_, arterial carbon dioxide tension.

Data presented as medians and interquartile ranges (IQR), numbers (percentage).

When simple weaning is the reference category, crude and adjusted odds ratios (ORs) for ICU, post-ICU, and hospital mortalities of the difficult and prolonged weaning groups in various statistical models are shown in Table 5. The prolonged weaning group had an OR of 5.327 (95% CI 3.069–9.249, *P* < 0.001) for hospital mortality in the crude state. In model 1, which was adjusted for demographic data, the prolonged weaning group had an adjusted OR of 5.332 (95% CI 2.963–9.595, *P* < 0.001) for hospital mortality. In model 2, which was additionally adjusted for causes of respiratory failure and severity of illness on ICU admission, a similar trend for hospital mortality of the prolonged weaning group (adjusted OR 5.501, 95% CI 3.000–10.085, *P* < 0.001) was maintained. Finally, in model 3, which additionally included the data on the day of first SBT, such as SOFA score, duration of MV before first SBT, weaning indices, and results of ABGA at the beginning and end of first SBT, the prolonged weaning group was still independently associated with hospital mortality (adjusted OR 5.528, 95% CI 2.801–10.910, *P* < 0.001). However, difficult weaning was not significantly associated with hospital mortality in the crude state or after adjustment for potential confounding factors. Similar trends of adjusted ORs for ICU and post-ICU mortality were observed (Table 5).

**Table 5:** Associations between the weaning classification and hospital mortality after adjustments for potential confounding factors.

		ICU mortality (n = 680)		Post-ICU mortality in ICU survivors (n = 618)		Hospital mortality (n = 680)	
Model	Weaning classification	OR (95% CI)	*P* value	OR (95% CI)	*P* value	OR (95% CI)	*P* value
Crude	Simple weaning	1.000 (Reference)		1.000 (Reference)		1.000 (Reference)	
	Difficult weaning	1.471 (0.586–3.696)	0.695	1.489 (0.861–2.575)	0.207	1.512 (0.920–2.485)	0.124
	Prolonged weaning	12.080 (5.960–24.473)	<0.001	2.540 (1.266–5.097)	0.005	5.327 (3.069–9.249)	<0.001
Model 1	Simple weaning	1.000 (Reference)		1.000 (Reference)		1.000 (Reference)	
	Difficult weaning	1.416 (0.555–3.612)	0.811	1.379 (0.778–2.444)	0.416	1.410 (0.840–2.368)	0.274
	Prolonged weaning	12.041 (5.715–25.369)	<0.001	2.546 (1.211–5.353)	0.010	5.332 (2.963–9.595)	<0.001
Model 2	Simple weaning	1.000 (Reference)		1.000 (Reference)		1.000 (Reference)	
	Difficult weaning	1.482 (0.572–3.842)	0.708	1.275 (0.710–2.289)	0.705	1.356 (0.797–2.306)	0.398
	Prolonged weaning	13.132 (6.009–28.698)	<0.001	2.593 (1.201–5.600)	0.011	5.501 (3.000–10.085)	<0.001
Model 3	Simple weaning	1.000 (Reference)		1.000 (Reference)		1.000 (Reference)	
	Difficult weaning	1.114 (0.403–3.077)	0.999	1.491 (0.780–2.851)	0.334	1.392 (0.780–2.484)	0.401
	Prolonged weaning	8.265 (3.484–19.605)	<0.001	3.180 (1.349–7.497)	0.005	5.528 (2.801–10.910)	<0.001

Model 1 was adjusted for demographic data, such as age, sex, and underlying disease.

Model 2 was additionally adjusted for the severity of illness on ICU admission (SAPS 3 and SOFA), and causes of respiratory failure

Model 3 was additionally adjusted for data on the day of first SBT, such as SOFA score, duration of MV before first attempt of SBT, weaning indices, PaCO_2_, and PaO_2_/FiO_2_ ratio at the beginning and end of first SBT.

ICU, intensive care unit; OR, odds ratio; CI, confidence interval; SAPS, simplified acute physiology score; SOFA, sequential organ failure assessment; MV, mechanical ventilation; SBT, spontaneous breath trial; PaCO_2_, arterial carbon dioxide tension; PaO_2_, arterial oxygen tension; FiO_2_, inspiratory oxygen fraction.

## Discussion

This study evaluated the prognostic significance of weaning classification based on the difficulty and duration of the weaning process. The results of our cohort study indicate that poor clinical outcomes, such as the need for tracheostomy, length of ICU/hospital stay, and high mortality increased significantly with weaning difficulty in our cohort of mechanically ventilated patients in a medical ICU with a standardized weaning program. Additionally, we found that prolonged weaning but not difficult weaning was independently associated with increased ICU, post-ICU, and hospital mortalities, even after adjustments for potential confounding factors.

From the international consensus conference on weaning from MV [6], approximately 69% of patients will be successfully extubated at the first weaning trial (simple weaning), while the remaining patients will experience difficult or prolonged weaning. However, the incidence of patients classified as simple weaning from recent reports was lower than that estimated at the conference, ranging instead from 30% to 55% [7–10]. In the present study, 67% of medical ICU patients who were mechanically ventilated for more than 24 hours and were weaned according to the standardized protocol were classified as simple weaning, consistent with the previous estimation [6]. The major difference in the incidence of simple weaning may be related to differing criteria for weaning readiness. In this study, all patients were screened daily for weaning readiness by RCPs based on the recent recommendations from the international consensus conference [6]. In addition, calculation of RSBI was also used as a final assessment of the readiness for SBT as in the recommendation [6]. Therefore, this study represents successful application of the recommendations and more accurately validates the prognostic significance of the weaning classification.

Prognostic significance of the weaning classification has been evaluated in the different type of ICUs [7–10]. Most studies evaluating the prognostic significance of the weaning classification with comparisons of outcomes among three groups tested the equality of proportions with multiple comparisons between pairs of groups. However, most of the previous studies could not find any differences in mortality between simple and difficult weaning groups [7–9], despite significant differences in the duration of MV before first weaning attempt [7, 8]. Significant differences in mortality were consistently observed in only the prolonged weaning group compared to simple weaning group [7–9]. In agreement with previous studies, differences in ICU and hospital mortality were observed between the three groups by the chi-square test (*P* < 0.001). In addition, increased ICU and hospital mortality was observed in patients with prolonged weaning in *post hoc* comparison to those with simple weaning (*P* < 0.001), but the difference did not reach statistical significance in *post hoc* comparison between simple and difficult weaning (*P* = 0.122), consistent with previous reports [7–9]. However, it has been suggested that the weaning classification be ordered by weaning difficulty and length of the weaning process. Therefore, trends of mortality across weaning groups should be tested to evaluate the clinical relevance of the classification. In this study, the trends of clinical outcomes across the weaning classification were evaluated with the Jonckheere-Terpstra test [16] and the Mantel–Haenszel test [17]. As a result, poor clinical outcomes, such as need for tracheostomy, increased length of stay, and high mortality, increased significantly across weaning groups. Therefore, the weaning classification may provide prognostic information for mechanically ventilated patients who underwent the weaning process.

There are other factors associated with poor clinical outcomes in mechanically ventilated patients who are subsequently weaned from MV, such as reason for MV [18] and duration of MV before first weaning attempt [19]. In addition, the total duration of MV is significantly associated with increased mortality [3]. Therefore, multiple logistic regression analysis was used to adjust for potential confounding factors in the association of hospital mortality across weaning groups in this study. After adjustment for severity of illness on ICU admission, causes of respiratory failure, and data from the day of first SBT, only prolonged weaning was significantly associated with increased ICU, post-ICU, and hospital mortalities compared with simple weaning, whereas the difficult weaning group had similar mortality to the simple weaning group, consistent with previous studies [7–9]. Therefore, delayed weaning that was finally successful within 1 week of the first attempt does not adversely influence mortality in mechanically ventilated patients [8].

There are several limitations to this study. First, given its retrospective nature, there is always the possibility that selection bias may have influenced the significance of our findings. However, the data were collected prospectively from all consecutive patients admitted to the medical ICU with MV support for more than 24 hours and screened daily for weaning readiness by a standardized weaning protocol. Secondly, our study was conducted at a single institution with a protocol-based weaning program, which may limit the generalizability of our findings to other centers in which no specific programs are available for weaning. Finally, a relatively large number of patients who underwent tracheostomy before the weaning process were excluded from the analysis. Tracheostomy is often considered in patients perceived as being difficult to wean [20]. Therefore, a high rate of tracheostomy may be associated with a lower proportion of prolonged weaning in this study compared to previous studies.

## Conclusions

Weaning classification based on the difficulty and duration of the weaning process may provide prognostic information for mechanically ventilated patients who undergo the weaning process, especially in patients with prolonged weaning.

## Supporting Information

S1 Table Considerations in assessing readiness for weaning. (DOCX)

S2 Table Criteria for spontaneous breathing trial (SBT) failure. (DOCX)

S3 Table Criteria for extubation failure within 48 hour after extubation. (DOCX)
