# Supplementary material for: Differences in Clinical Outcomes According to Weaning Classifications in Medical Intensive Care Units
Source: PLoS One. 2015 Apr 15;10(4):e0122810. doi: 10.1371/journal.pone.0122810 (PMC4398406; doi:10.1371/journal.pone.0122810)
Supplement: S1 Table — (DOCX) [file pone.0122810.s001.docx]

**Supporting Information** (Byeong-Ho Jeong et al.)

**Table S1. Considerations in assessing readiness for weaning.**

| Clinical assessment | Adequate cough  Absence of excessive tracheobronchial secretion  Resolution of disease acute phase for which the patient was intubated |
| --- | --- |
| Objective measurements | *Clinical stability*  Stable cardiovascular status (i.e. HR ≤ 120/min, SBP 90~140 mmHg, no or minimal vasopressors: dopamine ≤5mcg/kg/min, norepinephrine ≤0.05mcg/kg/min)  Stable metabolic status  *Adequate oxygenation*  SaO_2_ >90% on FiO_2_ ≤0.4 (or PF ratio > 200)  PEEP ≤5 cmH_2_O  *Adequate pulmonary function*  RR <35/min  Negative inspired pressure (NIP)^*^ ≤-15 cmH_2_O  V_T_ >5 mL/kg  V_E_ <10~15 L/min  RSBI [RR (in breaths/min) / V_T_ (in liters)] <105  No significant respiratory acidosis  *Adequate mentation*  No sedation or adequate mentation on sedation |

HR, heart rate; SBP, systolic blood pressure; SaO_2_, arterial oxygen saturation; FiO_2_, inspiratory oxygen fraction; PF ratio, arterial oxygen tension/inspiratory oxygen fraction ratio; PEEP, positive end expiratory pressure; RR, respiratory rate; V_T_, tidal volume; V_E_, minute ventilation; RSBI, rapid shallow breathing index.

^*^ Negative inspiratory pressure (NIP) is the lowest pressure generated during a forceful inspiratory effort against an occluded airway, which is determined by occluding the ventilator’s inspiratory port at the end of expiration for 20 seconds and reading the maximum negative pressure registered on the ventilator’s pressure manometer.
