# Supplementary material for: Differences in Clinical Outcomes According to Weaning Classifications in Medical Intensive Care Units
Source: PLoS One. 2015 Apr 15;10(4):e0122810. doi: 10.1371/journal.pone.0122810 (PMC4398406; doi:10.1371/journal.pone.0122810)
Supplement: S2 Table — (DOCX) [file pone.0122810.s002.docx]

**Supporting Information** (Byeong-Ho Jeong et al.)

**Table S2. Criteria for spontaneous breathing trial (SBT) failure.**

| Clinical assessment | Agitation and anxiety  Depressed mental status  Diaphoresis  Cyanosis  Evidence of increasing effort  Increased accessory muscle activity  Facial signs of distress  Dyspnea |
| --- | --- |
| Objective measurements | PaO_2_ <60 mmHg or SaO_2_ <90% on FiO_2_ ≥0.4  PaCO_2_ >45 mmHg or an increase in ≥20% from pre-SBT and pH <7.32 and a decrease in pH ≥0.07  RR >35/min or increased by ≥50%  HR >140/min or increased by ≥20%  SBP >180 mmHg or increased by ≥20%  SBP <90 mmHg  Cardiac arrhythmias |

PaO_2_, arterial oxygen tension; SaO_2_, arterial oxygen saturation; FiO_2_, inspiratory oxygen fraction; PaCO_2_, arterial carbon dioxide tension; SBT, spontaneous breathing trial; RR, respiration rate; HR, heart rate; SBP, systolic blood pressure.
