# Supplementary material for: Differences in Clinical Outcomes According to Weaning Classifications in Medical Intensive Care Units
Source: PLoS One. 2015 Apr 15;10(4):e0122810. doi: 10.1371/journal.pone.0122810 (PMC4398406; doi:10.1371/journal.pone.0122810)
Supplement: S3 Table — (DOCX) [file pone.0122810.s003.docx]

**Supporting Information** (Byeong-Ho Jeong et al.)

**Table S3. Criteria for extubation failure within 48 hour after extubation.**

| RR >25/min for 2 hour  HR >140/min or sustained increase or decrease of ≥20%  Clinical signs of respiratory muscle fatigue or increased work of breathing  Inadequate oxygenation: PaO_2_ <60 mmHg or SaO_2_ <90% on FiO_2_ ≥0.4  Inadequate ventilation: PaCO_2_ >45 mmHg or an increase in ≥20% from pre-extubation |
| --- |

RR, respiration rate; HR, heart rate; PaO_2_, arterial oxygen tension; SaO_2_, arterial oxygen saturation; FiO_2_, inspiratory oxygen fraction; PaCO_2_, arterial carbon dioxide tension
